# Alterations in the gut microbiota and metabolite profiles in the context of neuropathic pain

**DOI:** 10.1186/s13041-021-00765-y

**Published:** 2021-03-09

**Authors:** Peng Chen, Chen Wang, Yan-na Ren, Zeng-jie Ye, Chao Jiang, Zhi-bing Wu

**Affiliations:** 1 Basic Medical School, Guizhou University of Traditional Chinese Medicine, Guiyang, China; 2 First Clinical Medical School, Guangzhou University of Chinese Medicine, Guangzhou, China; 3 Department of Neurology, The Second Affiliated Hospital of Xi’an Medical University, Xi’an, China

**Keywords:** Neuropathic pain, Gut microbiota, Metabolite profiles, CCI model

## Abstract

The aim of this study was to explore the relationships among gut microbiota disturbances and serum and spinal cord metabolic disorders in neuropathic pain. 16S rDNA amplicon sequencing and serum and spinal cord metabolomics were used to identify alterations in the microbiota and metabolite profiles in the sham rats and the chronic constriction injury (CCI) model rats. Correlations between the abundances of gut microbiota components at the genus level, the levels of serum metabolites, and pain-related behavioural parameters were analysed. Ingenuity pathway analysis (IPA) was applied to analyse the interaction networks of the differentially expressed serum metabolites. First, we found that the composition of the gut microbiota was different between rats with CCI-induced neuropathic pain and sham controls. At the genus level, the abundances of *Helicobacter*, *Phascolarctobacterium*, *Christensenella*, *Blautia*, *Streptococcus*, *Rothia* and *Lactobacillus* were significantly increased, whereas the abundances of *Ignatzschineria*, *Butyricimonas*, *Escherichia*, *AF12*, and *Corynebacterium* were significantly decreased. Additionally, 72 significantly differentially expressed serum metabolites and 17 significantly differentially expressed spinal cord metabolites were identified between the CCI rats and the sham rats. Finally, correlation analysis showed that changes in the gut microbiota was significantly correlated with changes in serum metabolite levels, suggesting that dysbiosis of the gut microbiota is an important factor in modulating metabolic disturbances in the context of neuropathic pain. In conclusion, our research provides a novel perspective on the potential roles of the gut microbiota and related metabolites in neuropathic pain.

## Introduction

Neuropathic pain is defined as pain caused by a lesion or disease of the somatosensory nervous system and has a prevalence of 6.9–10% in the general population [1, 2]. Neuropathic pain may result from exposure to toxins or many different kinds of diseases, including metabolic diseases; neurodegenerative, vascular or autoimmune disorders; tumours; trauma; infection; and hereditary diseases [3]. Neuropathic pain is manifested as spontaneous or evoked pain, hyperalgesia (an increased pain response to a noxious stimulus), and allodynia (a painful response elicited by a normally non-nociceptive stimulus), which affect the health and life quality of patients. However, the mechanisms underlying neuropathic pain remain unclear, and the pharmacologic agents recommended as first-line treatments exhibit less than satisfactory analgesic effects [4, 5]. Therefore, a comprehensive understanding of the pathogenesis of neuropathic pain is urgently needed to develop more effective therapeutic strategies.

The adult human gut is colonized by a large variety of commensal microorganisms, which are collectively called the gut microbiota [6]. The gut microbiota plays an essential role in bidirectional communication between the gut and the central nervous system, including the brain and spinal cord, via immunological, hormonal and neuronal signals [7, 8]. Dysfunction of this bidirectional communication is involved in numerous diseases of the nervous system, such as Parkinson’s disease, Alzheimer's disease, stroke, multiple sclerosis and spinal cord injury [9–13]. Additionally, the gut microbiota has been reported to be a pivotal regulator that directly or indirectly mediates the development of neuropathic pain through a complex network of immune, metabolic, endocrine, and neural signalling pathways [14]. To date, the specific mechanisms underlying gut microbiota-mediated pain progression remain largely unknown.

The gut microbiota regulates several metabolic and neurological signalling pathways in the host that can be associated with neuropathic pain [14]. The spinal dorsal horn (SDH) is the primary centre for the processing and transmission of pain perception and plays an essential role in the initiation and maintenance of neuropathic pain. The chronic constriction injury (CCI) model, a classical model that was initially developed by Bennett and Xie, is the most widely applied model of peripheral nerve injury-induced neuropathic pain [15, 16]. In this study, we explored the relationship among gut microbiota disturbances and serum and spinal cord metabolic disorders in the CCI model to reveal the possible mechanism through which the gut microbiota mediates neuropathic pain 16S rDNA sequencing and emtaboloimcs.

## Materials and methods

### Animals

Sixteen 5- to 6-week-old male SD rats weighing 180–220 g (Hunan SJA Laboratory Animal Co., Ltd, SCXK (Hunan) 2016-0002) were housed in a specific pathogen-free (SPF) laboratory and randomly divided into the sham (control) group and CCI group (n = 8 in each group). As previously described in the literature, the rats were anaesthetized with pentobarbitone sodium and fixed to an operating table [16]. Then, the skin of the lower 1/3 of the left thigh was incised to expose the sciatic nerve. The nerve was ligated by making four knots with 4–0 silk thread at 1-mm intervals until the surrounding muscles twitched briefly. In the sham rats, the left sciatic was exposed but not subjected to any additional manipulation.

### Behavioural tests

Pain-related behavioural parameters, including the mechanical withdrawal threshold (MWT) and thermal withdrawal latency (TWL), were assessed the day before and on the 3rd, 7th, 11th, and 15th days after the operation.

To assess the MWT, the rats were placed in a transparent plexiglass box (22 cm × 12 cm × 22 cm) with a metal mesh floor for a 30-min adaptation period. An electronic von Frey anaesthesiometer (IITC Life Science Instruments, Woodland Hills, CA, USA) was used to stimulate the plantar surface of the left hind paw of each rat five times at five-minute intervals with increasing intensity. Lifting or licking of the paw indicated a positive response. The threshold on the display screen was recorded, and the average value was calculated as the MWT.

To assess the TWL, the rats were placed in a transparent plexiglass box (20 cm × 15 cm × 18 cm) with a glass plate on the bottom for a 30-min adaptation period. A radiant heat source (Model 390, IITC Life Science Instruments, Woodland Hills, CA, USA) was used to stimulate the plantar surface of the left hind paw of each rat three times at ten-minute intervals. When the rat lifted or licked its paw, the stop button was pressed, and the displayed time was recorded. The maximum irradiation time was 20 s to avoid burning. The average value was calculated as the TWL.

### Sample collection and preparation

All samples were collected after completion of the behavioural tests on the 15th postoperative day. Fresh faecal samples were collected in dry and clean tubes via abdominal massage. Each sample was frozen in liquid nitrogen and stored at − 80 °C for 16S rDNA amplicon sequencing. Then, rats were anaesthetized with pentobarbitone sodium. Ipsilateral spinal cord samples were collected and washed in polybutylene succinate (PBS). Blood samples were obtained from the abdominal aorta and centrifuged at 3000×*g* for 10 min to extract serum. The spinal cord and serum samples were frozen in liquid nitrogen and stored at − 80 °C for UPLC-Q-TOF/MS analysis.

### 16S rDNA amplicon sequencing

Total genomic DNA was extracted from each sample using the cetyltrimethylammonium bromide (CTAB) method and purified [17]. The DNA purity and concentration were measured using agarose gel electrophoresis. The V3-V4 region of the 16S rRNA gene was amplified by PCR using specific barcoded primers. PCR was performed in a 30 μL reaction, which included 15 μL Phusion® High-Fidelity PCR Master Mix (New England Biolabs, Ipswich, MA, USA), 0.2 μM forward and reverse primers, and 10 ng template DNA. The PCR products were analysed using 2% agarose gel electrophoresis, purified with an AxyPrepDNA Gel Extraction Kit (Axygen Bioscience, Union City, USA) and sequenced on an Illumina MiSeq/HiSeq 2500 platform to generate paired-end reads.

Operational taxonomic units (OTUs) were clustered with 97% similarity, and taxonomic information was annotated using the Ribosomal Database Project (RDP) classifier. Beta diversity analysis, including principal component analysis (PCA), principal coordinate analysis (PCoA) and weighted UniFrac distance, was performed with the Quantitative Insights into Microbial Ecology (QIIME) software package. Differences between groups were analysed using T-tests, linear discriminant analysis (LDA) effect size (LEFSe), and analysis of similarity (ANOSIM).

### Serum and spinal cord metabolomics

Serum (100 μL) and spinal cord (60 mg) samples were collected, mixed with adequate amounts of precooled acetonitrile/methanol (1:1, v/v), and centrifuged for 20 min at 4 °C and 14,000×*g* to collect the supernatant. For LC–MS analysis, the samples were separated using ultra-high-performance liquid chromatography (UHPLC, 1290 Infinity LC, Agilent Technologies, Santa Clara, CA, USA). Electrospray ionization (ESI) was used for detection in both positive and negative ion modes. Mass spectrometry analysis and metabolite identification were performed using an Agilent 6550 iFunnel Q-TOF spectrometer (Agilent Technologies, Santa Clara, CA, USA) and a Triple TOF 6600 mass spectrometer (SCIEX, Framingham, MA, USA), respectively.

The raw data were converted to the mzXML format using Proteowizard (http://proteowizard.sourceforge.net/) and imported into XCMS software for further analysis, including retention time correction, peak alignment and picking. Following Pareto-scaling preprocessing, the data were subjected to multivariate data analysis, including PCA, partial least square discriminant analysis (PLS-DA), orthogonal PLS-DA (OPLS-DA), and univariate statistical analysis, including fold change (FC) analysis and T-tests. Mean of metabolite concentrations in each group was used to calculate FC values. Differentially expressed metabolites were selected according to the following screening criteria: variable importance in projection (VIP) > 1 and P < 0.05.

Ingenuity pathway analysis (IPA) (www.ingenuity.com) was applied to analyse the interaction networks of the differentially expressed serum metabolites. Briefly, metabolite identifiers and corresponding FC expression data were exported to IPA software, and interaction networks with a score > 2 were displayed.

### Correlation analysis

Correlations between the abundances of gut microbiota components at the genus level, the levels of serum metabolites, and pain-related behavior were analysed. The correlation coefficients were calculated using the Spearman algorithm in R Version 3.4.2. A correlation network was constructed with Cytoscape Version 3.5.1.

### Statistical analysis

The behavioural data are expressed as the mean ± standard deviation and were statistically analysed by T-test using SPSS 21.0 software. P values of less than 0.05 were considered statistically significant.

## Results

### CCI-induced neuropathic pain results in behavioural changes

**Fig. 1:**
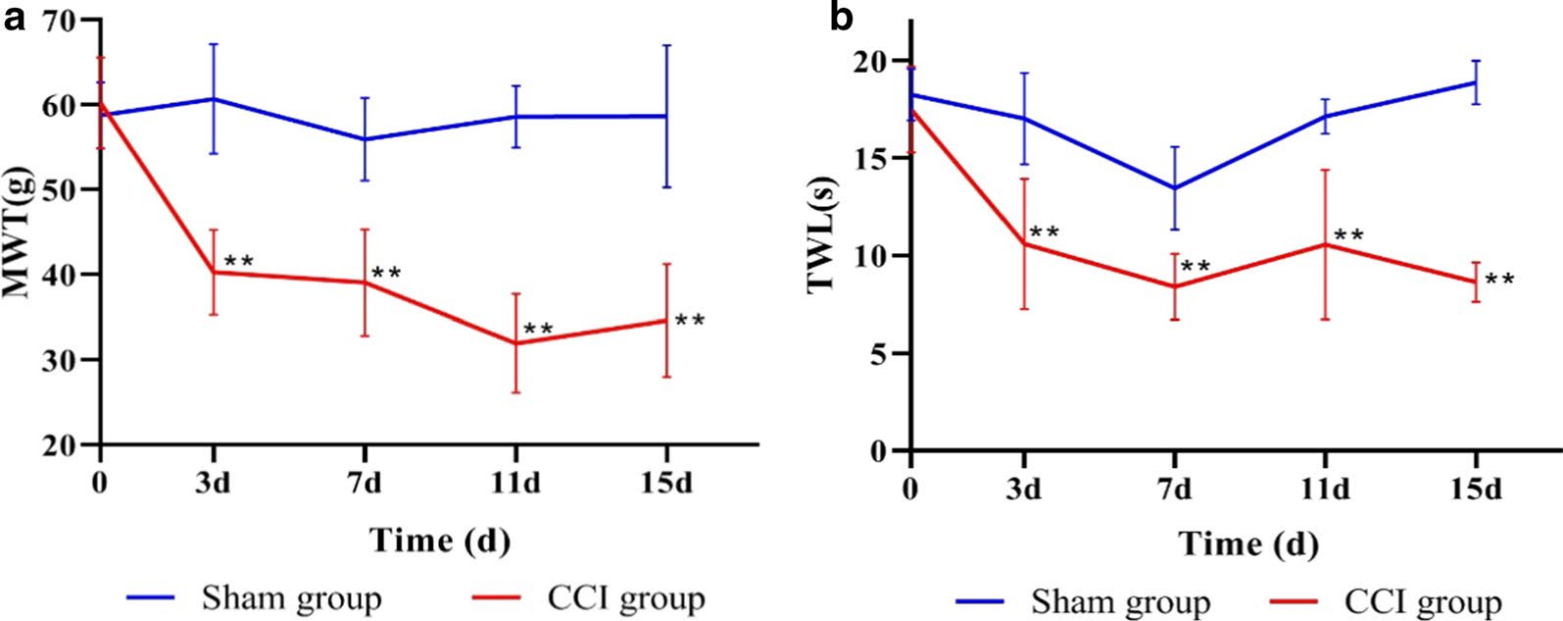
The MWT and TWL of the sham and CCI rats the day before and on the 3rd, 7th, 11th, and 15th days after the operation. **a** The MWT of the sham and CCI rats the day before and on the 3rd, 7th, 11th, and 15th days after the operation. **b** The TWL of the sham and CCI rats the day before and on the 3rd, 7th, 11th, and 15th days after the operation. **P < 0.01 versus the sham group

The pain-related behavioural parameters, including the MWT and TWL, of the sham and CCI groups were measured preoperatively and 3, 7, 11, and 15 days postoperatively. Compared with those of the sham group, the ipsilateral MWT and TWL of the CCI group were significantly reduced on the 3rd postoperative day and remained at a low level during the 15-day period of observation (P < 0.01). As shown in Fig. 1a and b, the rats in the CCI group displayed obvious hyperalgesia.

### CCI significantly alters the gut microbiota composition

**Fig. 2:**
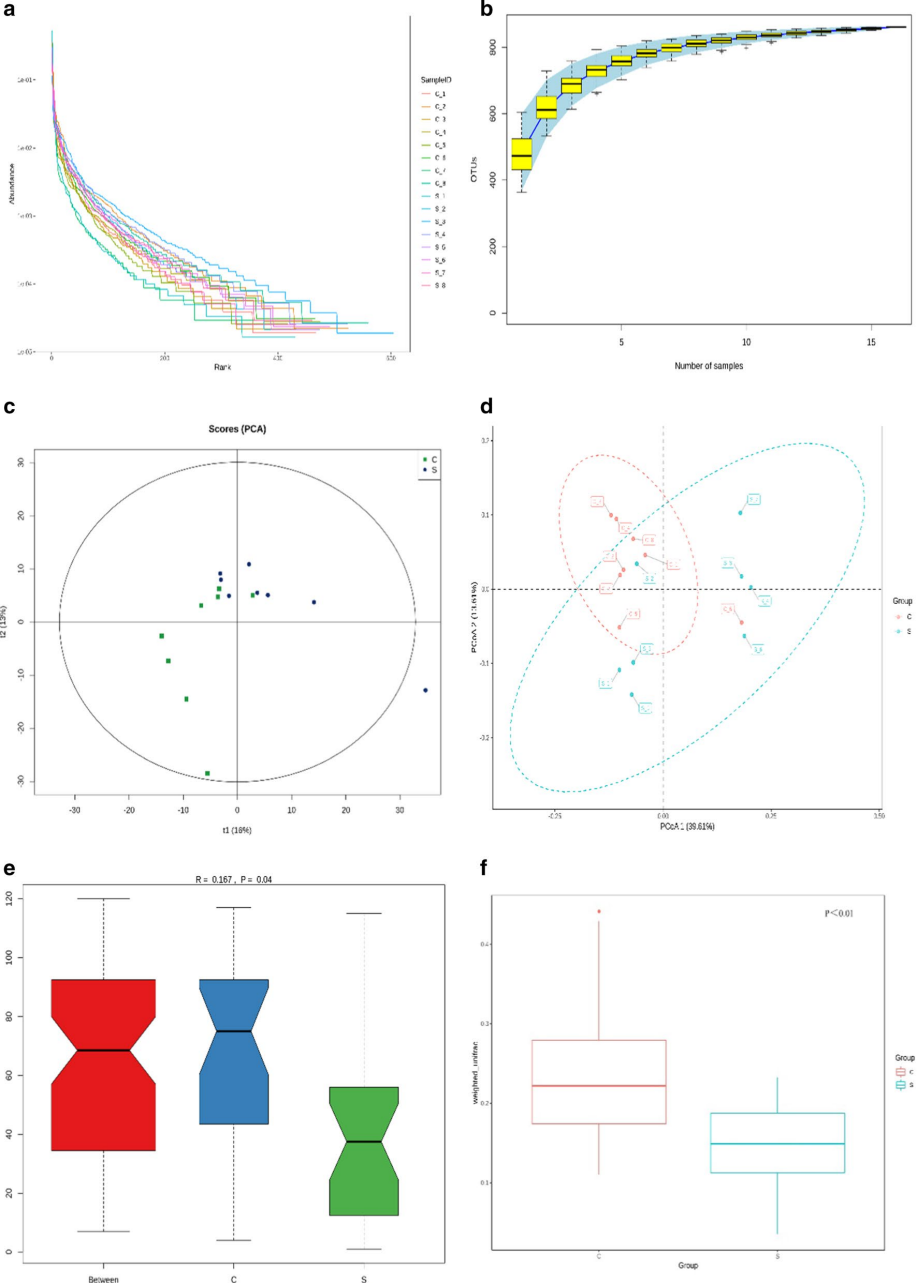
The alterations of the gut microbiota composition between the sham group (S) and CCI group (C). **a** Rank-abundance curves for different faecal samples from the sham and CCI groups. **b** Species accumulation curves. **c**–**f** PCA, PCoA, ANOSIM, and weighted UniFrac distances. (See figure on previous page)

To study whether the gut microbiota plays a role in CCI-induced neuropathic pain, 16S rDNA amplicon sequencing of faecal samples from the sham group (n = 8) and the CCI group (n = 8) was performed. The species accumulation curves of samples from the two groups were used to analyse the richness and evenness of the microbiological composition (Fig. 2a). The species accumulation curves indicated that the sample size was sufficient (Fig. 2b). Beta diversity analysis showed significant differences in gut microbiota composition between faecal samples from the sham and CCI groups. The PCA and PCoA results showed a clear separation of gut microbiota between rats from the sham and CCI groups (Fig. 2c, d). ANOSIM and weighted UniFrac analysis showed that the community structure and composition of the gut microbiota in the CCI group were different from those in the sham group, indicating that CCI induced dysbiosis of the gut microbiota (Fig. 2e, f).

**Fig. 3:**
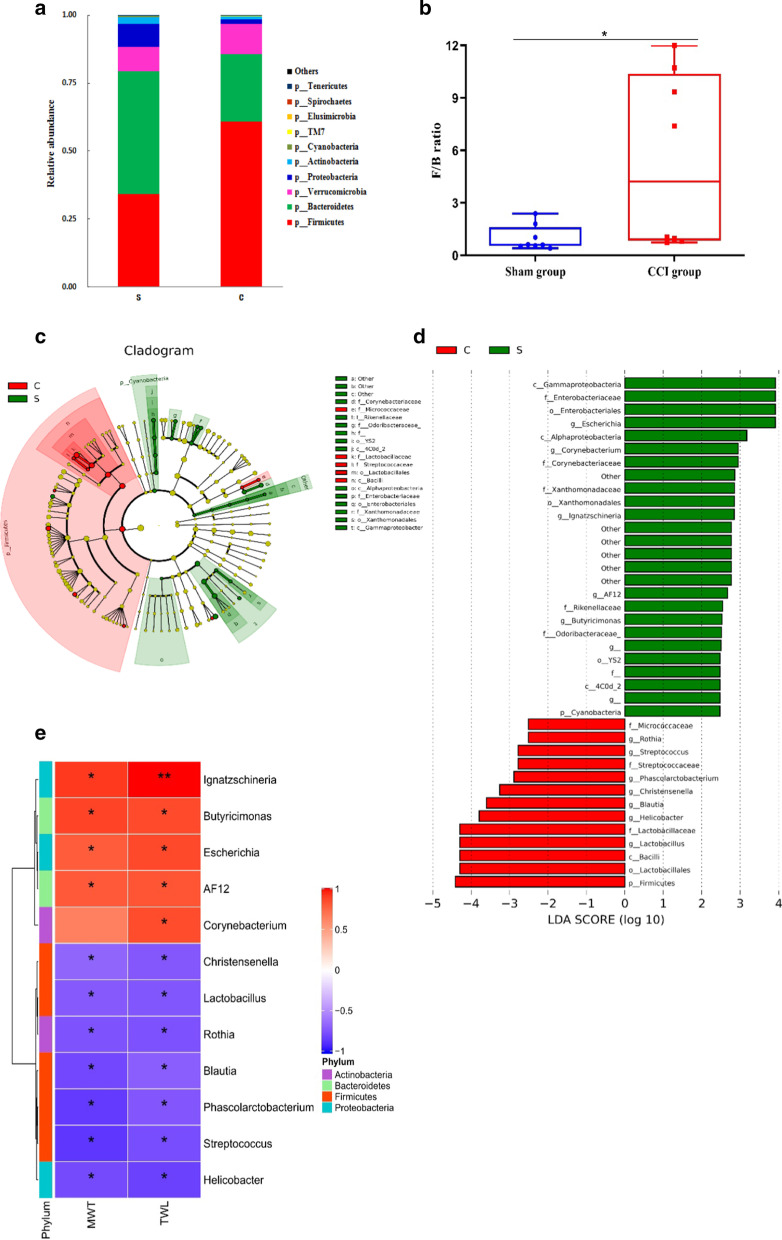
Alterations in gut microbiota phylotypes at the phylum and genus levels between the sham group (S) and CCI group (C). **a** The 10 species with the greatest abundance at the phylum level in the sham and CCI groups. **b** The F/B ratio in the sham and CCI groups. *P < 0.05 versus the sham group. **c**, **d** Histogram and cladogram of LEFSe analysis results in the sham and CCI groups. (E) Correlations between differential gut microbiota components at the genus level and pain-related behavioural parameters, including the MWT and TWL

Based on the results of species annotation, the 10 species with the greatest abundances in each group at different taxonomic levels (phylum, class, order, family, genus and species) were selected. At the phylum level, the abundances of *Firmicutes* and *Verrucomicrobia* were markedly increased, while the abundances of *Bacteroidetes* and *Proteobacteria* were significantly decreased in the intestines of the CCI rats (Fig. 3a). The *Firmicutes*/*Bacteroidetes* (F/B) ratio is considered an indicator of health status and is thought to reflect the degree of dysbiosis of the gut microbiota [18]. The F/B ratio in the CCI group was significantly higher than that in the sham group (Fig. 3b).

To identify the specific gut microbiota components associated with neuropathic pain, LEFSe analysis was used to differentiate the components of the gut microbiota of the CCI rats from those of the gut microbiota of the sham rats. LEFSe analysis revealed 39 distinguishing components at different taxon levels, including 13 species enriched in the CCI rats and 26 species enriched in the sham rats (LDA > 2, P < 0.05, Fig. 3c, d). At the phylum level, the 13 species enriched in the CCI group belonged to *Firmicutes* (n = 10), *Actinobacteria* (n = 2) and *Proteobacteria* (n = 1), while the 26 species enriched in the sham group belonged to *Proteobacteria* (n = 8), *Bacteroidetes* (n = 5), *Cyanobacteria* (n = 5), *Actinobacteria* (n = 2), *Firmicutes* (n = 1) and others (n = 5).

At the genus level, the abundances of *Lactobacillus* (LDA = 4.30, *P* = 0.036), *Helicobacter* (LDA = 3.79, *P* = 0.011), *Blautia* (LDA = 3.60, *P* = 0.012), *Christensenella* (LDA = 3.26, *P* = 0.035), *Phascolarctobacterium* (LDA = 2.89, *P* = 0.008), *Streptococcus* (LDA = 2.78, *P* = 0.005), and *Rothia* (LDA = 2.51, *P* = 0.045) were significantly increased in the gut microbiota of the CCI-induced rats. The results also showed obvious decreases in the abundances of *Escherichia* (LDA = 3.91, *P* = 0.009), *Corynebacterium* (LDA = 2.95, *P* = 0.011), *Ignatzschineria* (LDA = 2.85, *P* = 0.001), *AF12* (LDA = 2.68, *P* = 0.008), and *Butyricimonas* (LDA = 2.53, *P* = 0.002).

To explore the pathological significance of gut microbiota dysbiosis induced by CCI, the correlations between the abundances of the differential gut microbiota and pain-related behavioural parameters, including the MWT and TWL, were analysed using Spearman correlation analysis. The abundances of *Ignatzschineria*, *Butyricimonas*, *Escherichia* and *AF12* were positively correlated with the MWT and TWL, while the abundances of *Streptococcus*, *Phascolarctobacterium*, *Helicobacter*, *Blautia*, *Rothia*, *Lactobacillus* and *Christensenella* were negatively correlated with the MWT and TWL (Spearman r > 0.5 and P < 0.05) (Fig. 3e).

### CCI alters metabolism in the serum and spinal cord

CCI-induced neuropathic pain alters metabolism in the serum.

**Fig. 4:**
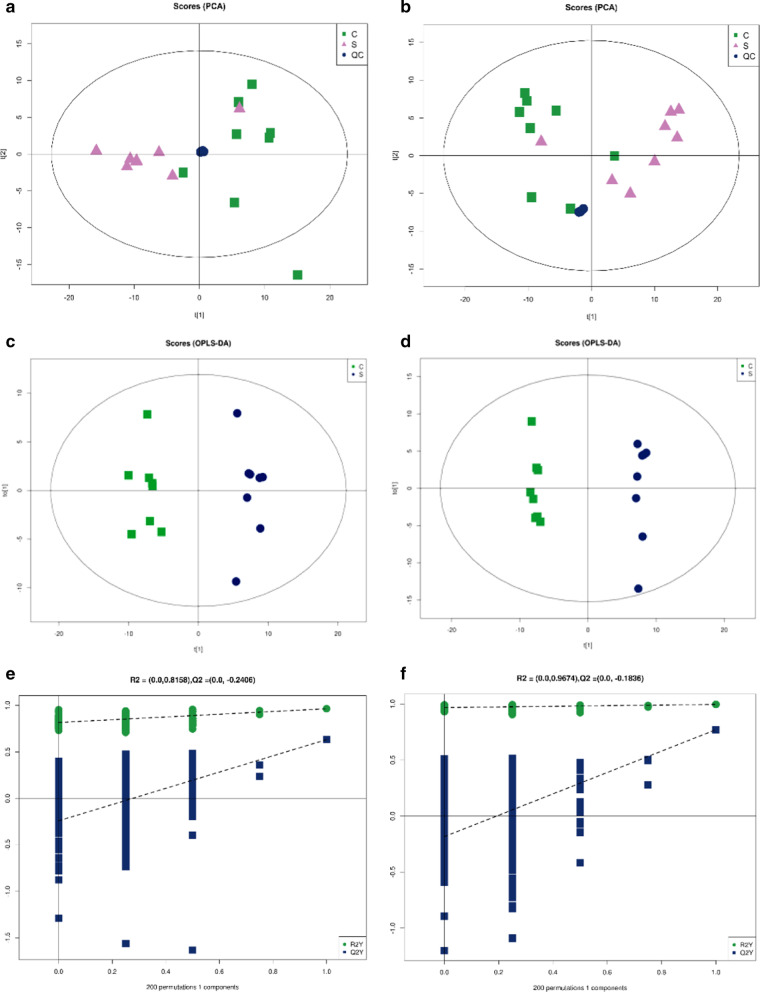
PCA score plots, OPLS-DA score plots and permutation tests in serum samples between the sham group (S) and CCI (C) group in positive and negative ion modes. **a**, **c**, **e** PCA score plots, OPLS-DA score plots and permutation tests between the sham and CCI groups in positive ion mode. **b**, **d**, **f** PCA score plots, OPLS-DA score plots and permutation tests between the sham and CCI groups in negative ion mode

To identify the metabolic disorders induced by CCI, untargeted metabolomics analysis was employed to evaluate the metabolic changes in serum samples between the sham group (n = 8) and the CCI group (n = 8). A total of 11,971 positive-mode features and 9460 negative-mode features were identified in the metabolic profiles of all samples. Multivariate statistics, including PCA, PLS-DA and OPLS-DA, were applied to analyse the data. An obvious separation between the sham rats and the CCI rats in the PCA and OPLS-DA score plot was shown, indicating that CCI induced severe metabolic dysfunction (Fig. 4a–d). Furthermore, 200 permutation tests showed that these patterns had good reliability (Fig. 4e, f).

**Fig. 5:**
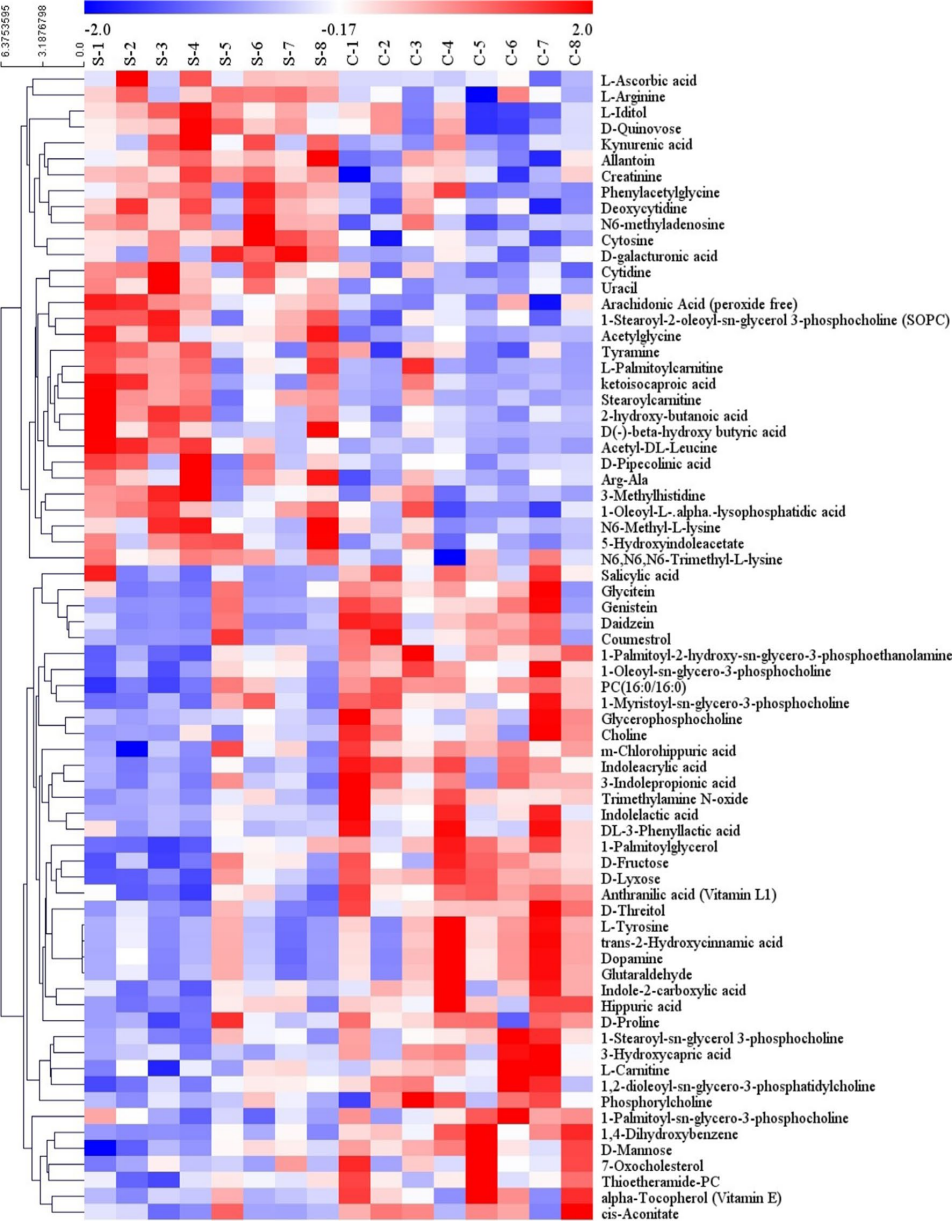
A clustered heat map displaying the trends in changes in the levels of significantly differentially expressed metabolites in serum samples from sham rats and CCI rats

From the OPLS-DA model, potential metabolites were identified based on specific screening conditions (VIP > 1 and P < 0.05). Seventy-two significantly differential metabolites, including 41 upregulated metabolites and 31 downregulated metabolites, were identified in the CCI rats compared with the sham rats (Table 1). A clustered heat map was used to visualize the trends of changes in significantly differentially expressed metabolites between the two groups (Fig. 5).

**Table 1:** Differentially expressed metabolites in the serum and spinal cord between the sham and CCI rats

Adduct	Metabolite	Serum			Spinal cord		
		VIP	FC	P-value	VIP	FC	P-value
(M + H) +	Anthranilic acid (Vitamin L1)	9.89	1.70	< 0.001	–	–	–
(M + H) +	Cytosine	5.53	0.85	< 0.001	–	–	–
(M + Na) +	PC (16:0/16:0)	3.60	1.41	0.001	–	–	–
(M + H) +	Uracil	1.17	0.61	0.002	–	–	–
(M + H–H_2_O) +	1**-**Palmitoylglycerol	2.37	1.70	0.002	1.19	0.50	0.042
(M + H) +	Creatinine	9.13	0.81	0.003	–	–	–
(M + H) +	Trimethylamine *N-*oxide	2.49	3.70	0.004	–	–	–
(M + Na) +	1**-**Stearoyl**-**2**-**oleoyl**-**sn**-**glycerol 3**-**phosphocholine (SOPC)	23.88	0.77	0.005	–	–	–
(M + H) +	1**-**Oleoyl**-**sn**-**glycero**-**3**-**phosphocholine	15.69	1.49	0.005	–	–	–
(M + H) +	Cytidine	1.87	0.59	0.005	–	–	–
(M + H) +	Deoxycytidine	2.23	0.92	0.006	–	–	–
(M + H) +	Daidzein	6.36	3.64	0.007	–	–	–
(M + H) +	l-Tyrosine	3.20	1.69	0.008	–	–	–
(M + H) +	N6**-**methyladenosine	1.52	0.79	0.008	–	–	–
(M + H) +	Trans**-**2**-**hydroxycinnamic acid	2.93	1.71	0.009	–	–	–
(M + H) +	Genistein	2.07	3.69	0.010	–	–	–
(M + H**-**H_2_O) +	Dopamine	2.56	1.68	0.010	–	–	–
(M + H) +	Stearoylcarnitine	1.23	0.45	0.010	–	–	–
M +	Choline	1.27	1.30	0.010	–	–	–
(M + Na) +	Glutaraldehyde	1.40	1.67	0.011	–	–	–
(M + H) +	l-Arginine	14.67	0.68	0.012	–	–	–
M +	Glycerophosphocholine	6.45	1.71	0.014	–	–	–
(M + H) +	Indole**-**2**-**carboxylic acid	1.32	2.04	0.014	–	–	–
M +	Arg**-**Ala	1.01	0.64	0.015	–	–	–
(M + H) +	1**-**Palmitoyl**-**sn**-**glycero**-**3**-**phosphocholine	5.06	1.30	0.015	–	–	–
(M + H) +	1,2**-**Dioleoyl**-**sn**-**glycero**-**3**-** phosphatidylcholine	9.77	1.98	0.016	–	–	–
(M + Na) +	1**-**Stearoyl**-**sn**-**glycerol 3**-**phosphocholine	1.03	1.38	0.018	–	–	–
(M + H) +	Kynurenic acid	1.01	0.53	0.020	–	–	–
(M + H) +	l-Carnitine	1.56	1.43	0.028	–	–	–
(M + H–H_2_O) +	N6**-**Methyl**-l-**lysine	1.50	0.43	0.033	–	–	–
(M + H) +	1**-**Myristoyl**-**sn**-**glycero**-**3**-**phosphocholine	2.96	1.44	0.038	–	–	–
(M + H) +	Glycitein	1.61	2.29	0.040	–	–	–
(M + H) +	Phosphorylcholine	2.97	1.37	0.041	–	–	–
M +	Alpha**-**tocopherol (vitamin E)	3.81	1.51	0.041	–	–	–
(M + H) +	l-Palmitoylcarnitine	2.30	0.52	0.042	–	–	–
(M + H) +	N6,N6,N6-trimethyl-l-lysine	1.60	0.76	0.043	2.61	0.35	0.022
(M + H) +	3**-**Methylhistidine	2.68	0.82	0.043	1.39	0.81	0.008
(M + H) +	d-Pipecolinic acid	1.88	0.59	0.044	–	–	–
(M + H) +	Phenylacetylglycine	1.24	0.46	0.044	–	–	–
(M + Na) +	Thioetheramide**-**PC	17.02	1.31	0.046	–	–	–
(M + H–H_2_O) +	Tyramine	4.32	0.82	0.047	–	–	–
(M + H) +	7**-**Oxocholesterol	1.58	1.69	< 0.05	–	–	–
(M + H) +	Betaine	1.78	1.41	0.05	1.04	1.22	0.048
(M + H) +	l-Histidine	2.32	0.80	0.26	4.78	0.70	0.035
(M + H) +	Nicotinamide	–	–	–	20.68	0.84	< 0.05
(M + H) +	S-Methyl-5′-thioadenosine	–	–	–	4.23	0.64	0.022
(M + H) +	Thr-Ala	–	–	–	1.07	0.60	0.043
(M + H) +	l-Anserine	–	–	–	1.23	0.69	0.037
(M + H–H_2_O) +	*N*-Acetyl-d-glucosamine 6-phosphate	–	–	–	1.12	0.26	< 0.05
(M–H)–	d-Lyxose	1.49	1.32	< 0.001	–	–	–
(M–H)–	Indoleacrylic acid	6.64	3.18	< 0.001	–	–	–
(M–H)–	1,4**-**Dihydroxybenzene	3.54	4.28	0.001	–	–	–
(M–H)–	Acetylglycine	1.01	0.46	0.001	–	–	–
(M–H)–	1**-**Palmitoyl**-**2**-**hydroxy**-**sn**-**glycero**-**3**-**phosphoethanolamine	4.63	1.52	0.001	–	–	–
(M + CH_3_COO)–	d-Threitol	2.15	1.54	0.001	–	–	–
(M–H)–	Acetyl-dl-leucine	1.16	0.43	0.001	–	–	–
(M + CH_3_COO)–	d-Mannose	2.34	1.42	0.004	–	–	–
(M–H)–	Arachidonic acid (peroxide-free)	8.34	0.62	0.005	–	–	–
(M + Na–2H)–	3**-**Hydroxycapric acid	2.81	2.76	0.006	–	–	–
(M–H_2_O–H)–	d-Galacturonic acid	5.15	0.68	0.006	–	–	–
(M–H)–	3**-**Indolepropionic acid	14.43	2.39	0.008	–	–	–
(M–H)–	Hippuric acid	4.32	2.11	0.009	–	–	–
(M + Na–2H)–	1**-**Oleoyl-l-alpha**-**lysophosphatidic acid	1.32	0.76	0.011	–	–	–
(M–H)–	5-Hydroxyindoleacetate	1.77	0.68	0.012	–	–	–
(M–H)–	Allantoin	6.06	0.83	0.012	1.40	0.79	0.016
(M–H_2_O–H)–	l-Iditol	1.15	0.76	0.013	–	–	–
(M–H)–	2-Hydroxybutanoic acid	1.75	0.44	0.013	0.99	0.52	0.019
(2M–H)–	d-Fructose	1.88	1.41	0.014	–	–	–
(M–H)–	Indolelactic acid	1.54	2.01	0.015	–	–	–
(M + CH_3_COO)–	d-Quinovose	1.25	0.79	0.016	1.09	0.68	0.001
M–	M**-**chlorohippuric acid	4.33	1.47	0.020	–	–	–
(M–H)–	dl-3**-**phenyllactic acid	3.04	2.65	0.025	–	–	–
(M + Na**-**2H)–	l-Ascorbic acid	1.86	0.61	0.030	–	–	–
M–	Coumestrol	7.13	3.22	0.031	–	–	–
(M–H)–	Cis**-**aconitate	1.84	2.22	0.032	–	–	–
(M–H)–	Salicylic acid	1.05	2.09	0.032	–	–	–
(M–H)–	d(–)**-**beta**-**hydroxybutyric acid	4.35	0.33	0.032	1.12	0.43	0.032
(M–H)–	Ketoisocaproic acid	8.84	0.64	0.042	–	–	–
(M–H)–	d-Proline	1.79	1.18	0.047	–	–	–
(M–H)–	Citrate	1.58	1.66	0.297	13.77	2.10	0.005
(M–H)–	dl-Lactate	–	–	–	1.98	1.51	0.024
(M–H)–	3-Phosphoserine	–	–	–	1.83	2.69	0.034
(M–H)–	*N*-Acetylneuraminic acid	–	–	–	4.19	0.89	0.042

**Fig. 6:**
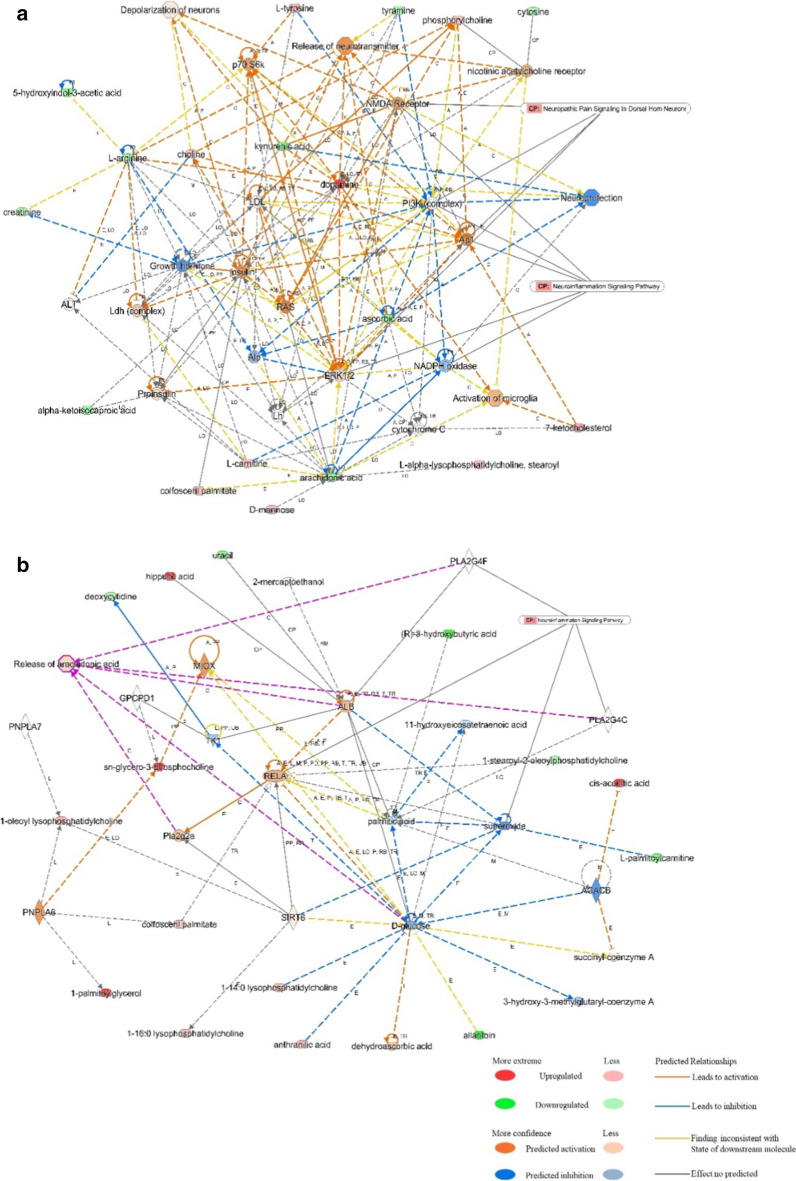
IPA-based interaction networks for the differentially expressed serum metabolites. **a** The free radical scavenging, lipid metabolism, and molecular transport interaction network. **b** The immunological disease, inflammatory disease, and inflammatory response interaction network

IPA software was applied to analyse the interactions between the differentially expressed metabolites (Table 2). The results showed that these differentially expressed serum metabolites were involved in several molecular and cellular functions and were specifically enriched in these two categories. The interaction network with the top score was free radical scavenging, lipid metabolism, and molecular transport, with 18 focus molecules being involved in several canonical pathways and biological functions, including neuropathic pain signalling in dorsal horn neurons, neuroinflammation signalling pathway, release of neurotransmitters, and depolarization (Fig. 6a). The interaction network with the second highest score was immunological disease, inflammatory disease, and the inflammatory response, with 15 focus molecules being involved in the neuroinflammatory signalling pathway and release of arachidonic acid (Fig. 6b).

**Table 2:** Interaction networks of the differential serum metabolites

ID	Score	Focus molecules	Top diseases and functions
1	45	18	Free radical scavenging, lipid metabolism, and molecular transport
2	36	15	Immunological disease, inflammatory disease, and inflammatory response
3	11	6	Cell death and survival, increased levels of LDH, and nervous system development and function
4	3	1	Nervous system development and function, neurological disease, and ophthalmic disease

CCI-induced neuropathic pain alters metabolism in the spinal cord.

**Fig. 7:**
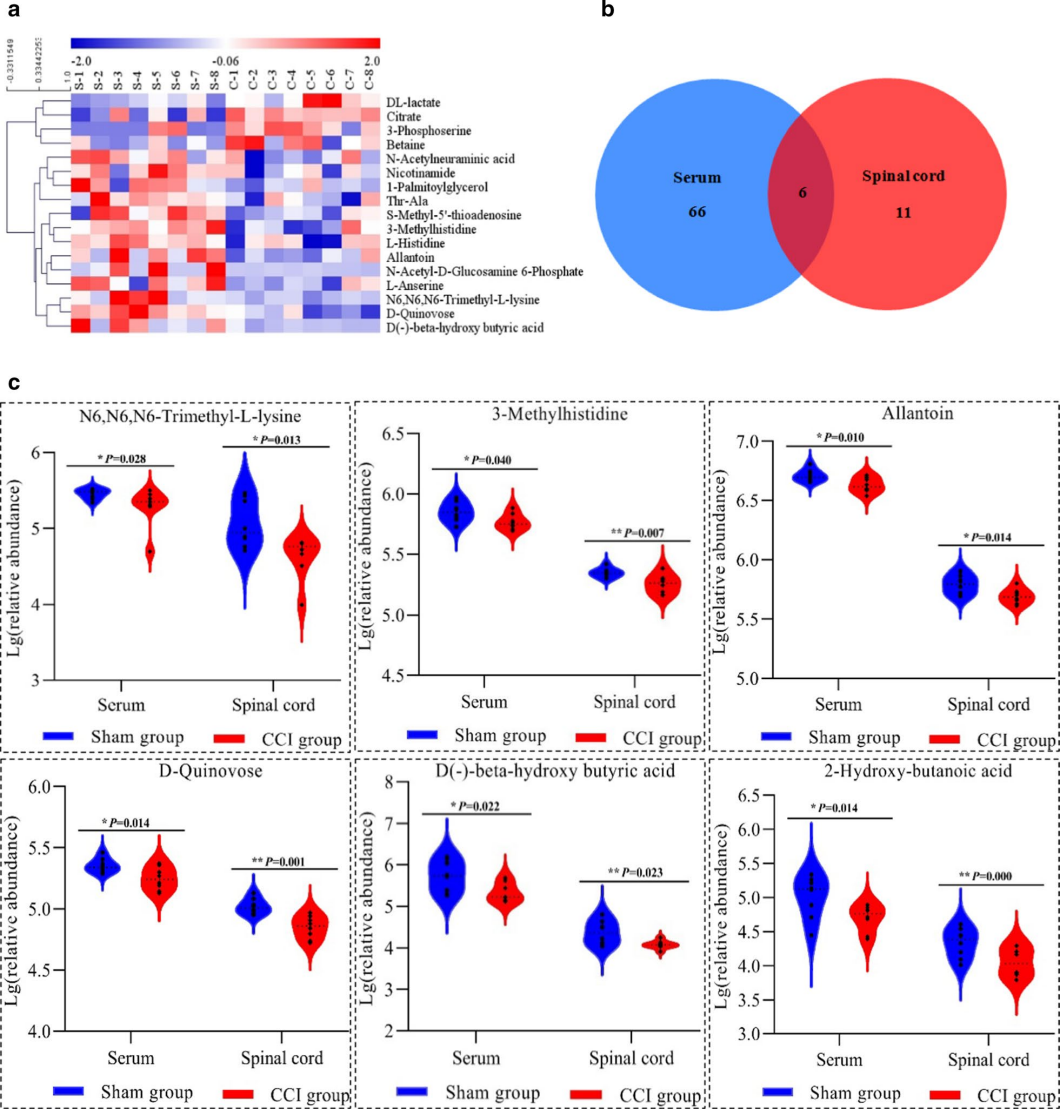
Differentially expressed metabolites in spinal cord samples and differentially expressed metabolites in both serum and spinal cord samples from the sham rats and the CCI rats. **a** A clustered heat map displayed the trends in the changes in significantly differentially expressed metabolites in spinal cord samples from the sham and CCI rats. **b** Venn diagram of differentially expressed metabolites in serum and spinal cord samples from the sham and CCI rats. **c** Differentially expressed metabolites in both serum and spinal cord samples from the sham and CCI rats

Untargeted metabolomics analysis was further used to identify metabolic dysfunction in the spinal cord induced by CCI. A total of 11,978 positive-mode features and 9218 negative-mode features were identified in the metabolic profiles of spinal cord samples from the sham rats and the CCI rats. PCA, PLS-DA and OPLS-DA were used to assess the variability between the two groups. In the OPLS-DA model, 17 significantly differential metabolites with the criteria of VIP > 1 and P < 0.05, including 4 upregulated metabolites and 13 downregulated metabolites, were identified in the CCI rats compared with the sham rats (Table 1, Fig. 7a).

Six common metabolites were identified by intersecting the differentially expressed metabolites in serum and spinal samples from the sham and CCI rats (Fig. 7b). Among these differentially expressed metabolites, 5, including N6,N6,N6**-**trimethyl**-l-**lysine, 3**-**methylhistidine, allantoin, d-quinovose and d(**–**)**-**beta**-**hydroxy butyric acid (BHB), exhibited consistent expression trends (Fig. 7c). In addition, 2-hydroxybutanoic acid (2-HB) was found at lower concentrations in both serum and spinal cord samples from CCI rats than those from sham rats, although the VIP values were slightly less than 1 (Fig. 7c).

### Correlation of differentially abundant gut microbiota components and the levels of serum metabolites

**Fig. 8:**
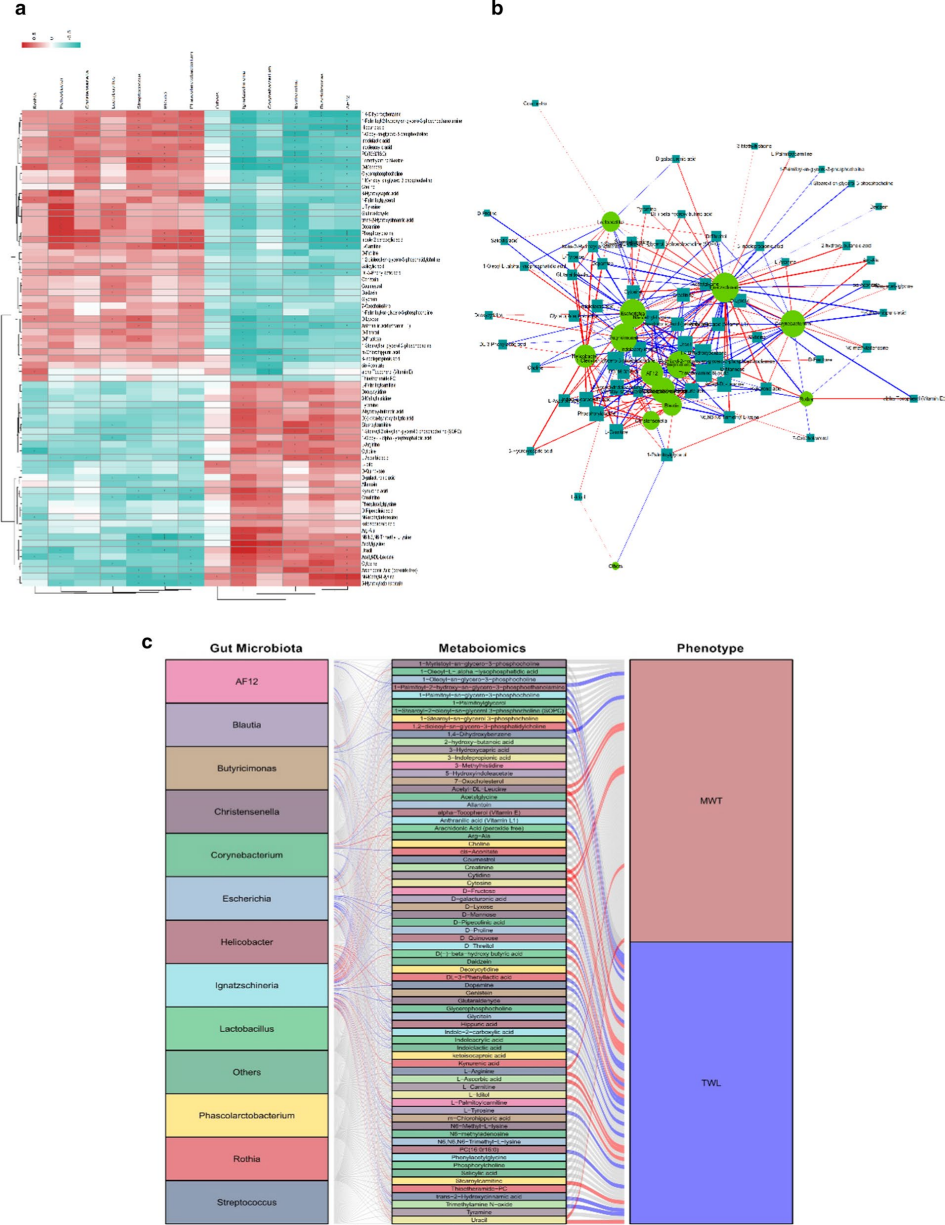
Correlation of gut microbiota disturbances, serum metabolite levels, and pain-related behavioural parameters. **a** and **b** A clustered heat map and network map of the correlations between the abundances of gut microbiota components and serum metabolite levels. **c** The correlations between gut microbiota disturbance, differential serum metabolites, and pain-related behavioural tests

To further study the functional significance of the metabolic dysfunction induced by gut microbiota disturbances in CCI rats, Spearman correlation analysis was used to calculate the association between the levels of 12 differentially abundant gut bacteria at the genus level and the levels of 73 differentially expressed metabolites in serum samples. Spearman correlation coefficients were visualized using a cluster heat map, with blue/green and red representing negative and positive correlations, respectively (|r|> 0.5 and P < 0.05, Fig. 8a, b). For example, the level of N6,N6,N6-trimethyl-l-lysine was significantly positively correlated with the abundances of *Ignatzschineria* and *AF12* but negatively correlated with the abundances of *Blautia*, *Streptococcus*, and *Phascolarctobacterium*. In addition, we performed correlation analysis of gut microbiota disturbances, the levels of serum metabolites, and pain-related behavioural parameters (|r|> 0.5 and P < 0.05, Fig. 8c). For instance, the D(−)-BHB level was positively correlated with the TWL and the abundances of *Butyricimonas* and *Ignatzschineria*.

## Discussion

In recent years, several studies have revealed that the gut microbiota is a crucial modulator of the pathogenesis of neuropathic pain. It was found that in a mouse model of chemotherapy-induced peripheral neuropathy (CIPN), the abundance of *Akkermansia muciniphila* was decreased, promoting barrier dysfunction and systemic inflammation and subsequently driving pain sensitivity [19]. Ding et al. reported that changes in the gut microbiota attenuated the development of CCI-induced neuropathic pain by modulating proinflammatory and anti-inflammatory T cells [20]. However, the detailed characteristics of the gut microbiota in the context of neuropathic pain remains largely unknown. In this study, we revealed changes in the gut microbiota composition in a classical CCI model and the potential mechanism of metabolic dysfunction mediated by the gut microbiota using 16S rDNA and metabolomics sequencing.

We demonstrated that the characteristics of the gut microbiota were changed in rats with CCI-induced neuropathic pain compared with sham controls. The F/B ratio is considered a representative indicator of health status and reflects the degree of gut microbiota dysbiosis. An increased F/B ratio was previously found to be associated with numerous nervous system diseases, such as stroke, cognitive impairments and sleep deprivation [21–23]. In our research, the F/B ratio was significantly increased in CCI rats, and this increase corresponded to the status of neuropathic pain. We confirmed that within the phylum *Firmicutes*, the abundances of *Phascolarctobacterium*, *Christensenella*, and *Blautia*, which belong to the order *Clostridiales*, were increased in the CCI group. These bacteria have been reported to be implicated in other neurological and psychiatric disorders [24–26]. In addition, several pathogenic bacteria, including *Streptococcus* and *Helicobacter*, were enriched in the CCI rats, and the abundances of these bacteria were significantly negative correlated with the MWT and TWL. We also found that *Ignatzschineria* and *Butyricimonas* were enriched in sham rats and that the abundances of these bacteria were positively correlated with the MWT and TWL. These results are essential for further studies investigating the key role of the gut microbiota in neuropathic pain progression.

The gut microbiota regulates a series of key metabolic functions of the body, and dysbiosis of the gut microbiota is an important factor in modulating host metabolic disturbances [27]. The result of the present study confirmed significant differences in metabolite levels in the serum and spinal cord between sham and CCI rats, suggesting that neuropathic pain may involve extensive metabolic disturbances. In our study, 72 differentially expressed serum metabolites, primarily those that regulate lipid metabolism and the inflammatory response, were identified in CCI rats. Seventeen differentially expressed metabolites, mainly those that mediate amino acid metabolism and energy production, were identified in spinal cord samples from CCI rats. We primarily focused on metabolites with consistent expression trends in serum and spinal samples and their relationship with the gut microbiota.

The abundances of *Ignatzschineria* and *Butyricimonas* were decreased in CCI rats, and these changes had a significant positive effect on the MWT and TWL. *Butyricimonas* is a butyrate-producing genus, and butyrate is a short-chain fatty acid with anti-inflammatory and anti-oxidative stress activities [28]. Recently, it was reported that a reduction in the abundance of *Butyricimonas* induces reductions in butyrate levels and promotes neuroinflammation in some nervous system diseases, such as multiple sclerosis, Parkinson’s disease, and depression [29–31]. We found that the abundance of *Butyricimonas* was positively correlated with the level of BHB. BHB is one of the most abundant ketone bodies and is primarily synthesized in the liver via the oxidation of fatty acids [32]. BHB is not only a passive energy carrier but also a key signalling molecule that participates extensively in neuronal function, lipid metabolism, and gene expression [32, 33]. The ketogenic diet is increasingly being used to treat neurological disorders, including seizures, Alzheimer’s disease, Parkinson’s disease, ischaemic stroke, and chronic pain [34–38]. We confirmed that CCI brought about a synchronous reduction in BHB levels in both the serum and spinal cord. Recently, it was also reported that BHB significantly relieves mechanical and thermal allodynia and improves locomotor function in mice with spinal cord injury, possibly by suppressing histone deacetylation and NLRP3 inflammasome activation and protecting mitochondrial function [39]. In addition, BHB can increase the concentration of gamma-aminobutyric acid (GABA), which is the main inhibitory neurotransmitter in the central nervous system and contributes to neuropathic pain [40, 41]. Therefore, we hypothesized that *Butyricimonas* might play a role in the development of neuropathic pain via ketone body metabolism.

*Ignatzschineria* species are aerobic, rod-shaped, non-spore-forming, gram-negative bacteria belonging to the phylum *Proteobacteria* [42]. At present, the roles of *Ignatzschineria* in human diseases are less reported than those of other bacteria and are underreported to some degree since phenotypic identification is challenging [43, 44]. Correlation analysis revealed that the abundance of *Ignatzschineria* was positively correlated with the levels of BHB, 3**-**methylhistidine, 2-HB, and N6,N6,N6-trimethyl-l-lysine, differentially expressed metabolites in the serum and spinal cord with the same decreasing tendencies. 3-Methylhistidine is one of the major l-histidine derivatives formed by the post-translational methylation of l-histidine residues [45]. In addition to that of 3-methylhistidine, the expression of l-histidine and l-anserine, which are involved in histamine metabolism, was significantly reduced in the spinal cords of CCI rats. l-Histidine is a histamine precursor and is decarboxylated by the enzyme histidine decarboxylase to generate histamine, which acts as an important neurotransmitter to regulate a series of biological functions of the nervous system. Histamine exerts disparate effects via distinct receptor subtypes (H1-4) with different pharmacological and signal transduction properties [46]. For example, presynaptic and post synaptic activation of the H3 receptor can reduce neuronal excitability, inhibit neuronal inflammation and produce pain relief [46]. Several studies have demonstrated the analgesic roles of histamine in the central nervous system in multiple rodent models of neuropathic pain [47–49]. 2-HB is an organic acid derived from α-hydroxybutyrate, which is an early marker of insulin resistance and impaired glucose regulation [50]. In our study, we found that the level of 2-HB was significantly decreased in both the sera and spinal cords of CCI rats, which might represent an adaptive response to lipid oxidation or oxidative stress [51, 52]. N6,N6,N6-trimethyl-l-lysine is a methylated derivative of lysine that serves as the substrate for carnitine biosynthesis. Therefore, we hypothesized that *Ignatzschineria* is closely related to histamine metabolism, ketone body metabolism and carnitine biosynthesis in neuropathic pain progression.

In addition, several pathogenic bacteria were enriched in the CCI rats, and the abundances of these bacteria were significantly negatively correlated with the MWT and TWL. *Helicobacter*, a specific genus of *Proteobacteria*, is involved in the occurrence and development of some nervous system diseases via multiple mechanisms, including neurotoxicity, neuroinflammation, microelement deficiency and so on [53, 54]. Our study showed that the abundance of *Helicobacter* was markedly increased in CCI rats and significantly positively associated with the levels of l-tyrosine and dopamine. The levels of several important metabolites involved in tyrosine metabolism, including l-tyrosine, dopamine and 1,4-dihydroxybenzene, were significantly elevated in the sera of CCI rats. l-tyrosine is the precursor of dopamine, and the latter is a key neurotransmitter in pain transmission [55]. *Streptococcus* is associated with inflammatory pain and some neurotransmitters, such as serotonin [56, 57]. In our research, the abundance of *Streptococcus* was found to be positively correlated with the level of anthranilic acid and negatively associated with the level of kynurenic acid. Anthranilic acid and kynurenic acid are important metabolic products of l-tryptophan.

## Conclusions

This study provides a novel perspective on the potential roles of the gut microbiota and related metabolites in neuropathic pain. The function and specific mechanism of the gut microbiota in neuropathic pain are extremely sophisticated. However, the limitations of this study need to be considered for future studies. First, faecal microbiota transplantation could be used to further verify the roles of the specific bacteria in neuropathic pain. Second, our studies were carried out in animal models, and the results require confirmation in the patients. Despite these limitations, our study makes a number of contributions to the field.

## Data Availability

Data openly available in a public repository that issues datasets with DOIs.
